# Whole-Genome Sequencing Confirms that Burkholderia pseudomallei Multilocus Sequence Types Common to Both Cambodia and Australia Are Due to Homoplasy

**DOI:** 10.1128/JCM.02574-14

**Published:** 2014-12-18

**Authors:** Birgit De Smet, Derek S. Sarovich, Erin P. Price, Mark Mayo, Vanessa Theobald, Chun Kham, Seiha Heng, Phe Thong, Matthew T. G. Holden, Julian Parkhill, Sharon J. Peacock, Brian G. Spratt, Jan A. Jacobs, Peter Vandamme, Bart J. Currie

**Affiliations:** a Department of Clinical Sciences, Institute of Tropical Medicine, Antwerp, Belgium; b Global and Tropical Health Division, Menzies School of Health Research, Darwin, Australia; c Imperial College, London, United Kingdom; d Sihanouk Hospital Centre of HOPE, Phnom Penh, Cambodia; e Institut Pasteur du Cambodge, Phnom Penh, Cambodia; f Laboratory of Microbiology, Faculty of Sciences, Ghent University, Ghent, Belgium; g Wellcome Trust Sanger Institute, Cambridge, United Kingdom; h University of St. Andrews, St. Andrews, United Kingdom; i University of Cambridge, Cambridge, United Kingdom; j Department of Microbiology and Immunology, University of Leuven, Leuven, Belgium

## Abstract

Burkholderia pseudomallei isolates with shared multilocus sequence types (STs) have not been isolated from different continents. We identified two STs shared between Australia and Cambodia. Whole-genome analysis revealed substantial diversity within STs, correctly identified the Asian or Australian origin, and confirmed that these shared STs were due to homoplasy.

## TEXT

Burkholderia pseudomallei, the bacterium that causes melioidosis, is well-recognized in the regions of northern Australia and Southeast Asia where melioidosis is prevalent. The prevalence of B. pseudomallei is increasingly being recognized in other tropical regions including parts of Africa, the Americas, and other Asian regions such as India (1). Melioidosis is a potentially fatal disease with mortality rates ranging from 10 to 50% of infected individuals (2). The majority of B. pseudomallei infections are acquired from the environment following percutaneous inoculation, inhalation, or ingestion of contaminated soil or surface water; human-to-human transmission is exceedingly rare (2). The nature of melioidosis acquisition, coupled with restricted B. pseudomallei environmental dissemination patterns, has contributed to the evolution of localized genetic populations with finite geographic distribution (3–5). Phylogeographic studies using multilocus sequence typing (MLST) (6) and whole-genome sequencing (4) have identified two distinct populations of B. pseudomallei corresponding to Asia and Australia (3, 4, 7, 8). Knowledge of this population structure has facilitated source attribution for unusual melioidosis cases, particularly those occurring in regions where melioidosis is not endemic, such as returning travelers (9, 10). Despite the success of previous studies in identifying robust phylogeographic patterns within B. pseudomallei populations, the inherently high recombination rate of this bacterium and greater sampling efforts were predicted to inevitably reveal shared sequence types (STs) between these distinct geographic locations (11). In the present study, we identify for the first time two such instances of B. pseudomallei STs being shared between Cambodian and Australian isolates.

B. pseudomallei sequence type 105 (ST105) and ST849 isolates were analyzed from both Australia and Cambodia; a total of four isolates were analyzed. MLST was performed as previously described (6). MSHR282, the Australian ST105 isolate, was obtained in 1994 from an Australian patient enrolled in the Darwin Prospective Melioidosis Study (12), and the Cambodian ST105 isolate, CAM41, was isolated from a Cambodian melioidosis patient in 2008. To date, no other ST105 isolates have been submitted to the MLST database (http://bpseudomallei.mlst.net/). MSHR4004, the Australian ST849 isolate, was isolated from an Australian soil sample in 2010, and the Cambodian ST849 isolate, SHCH2430, was isolated from a Cambodian melioidosis patient in the same year. Clinical isolates were from patients with strong epidemiological data to support local acquisition of their infections, including no documented travel history to other regions where melioidosis is endemic. Ethics were approved by the Human Research Ethics Committee of the Northern Territory Department of Health and Families, the Menzies School of Health Research, and the Cambodian National Ethical Committee.

**FIG 1:**
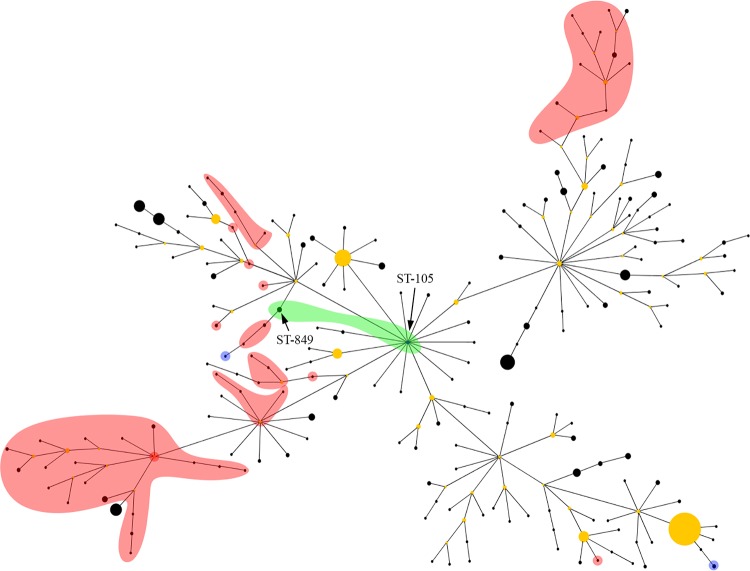
eBURST analysis of 954 Burkholderia pseudomallei isolates from Australia and Cambodia, comprising 245 sequence types (STs). Both ST105 and ST849 (shaded green) contain B. pseudomallei isolates found in Cambodia and Australia. All other STs have been found solely in Australasia or Asia. STs shaded in red indicate an Asian origin and include isolates found in Cambodia, Thailand, China, and Vietnam. STs shaded in blue represent islands in Australasia. All unshaded STs are of Australian origin.

The MLST profiles of all Cambodian and Australian B. pseudomallei strains in the MLST database (as of 13 June14) were first analyzed with eBURST V3 (13) (Fig. 1). eBURST showed some evidence of ST clustering according to geographic source; however, these groupings contained multiple cases of Asian isolates grouping with isolates of Australian origin (Fig. 1). Overall, eBURST was unreliable for inferring the geographic origin of STs, most likely due to very high rates of recombination in B. pseudomallei, which has previously been shown to confound accurate prediction of recent ancestry using this tool (11).

**FIG 2:**
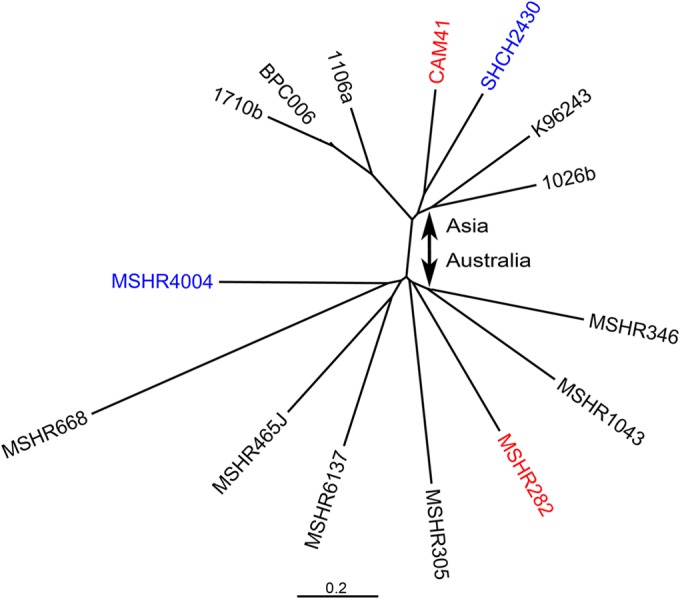
Maximum likelihood phylogenetic analysis of two Cambodian (CAM41 and SHCH2430) and two Australian (MSHR282 and MSHR4004) Burkholderia pseudomallei genomes in comparison to B. pseudomallei reference genomes. A total of 84,839 core genome single-nucleotide polymorphisms were used to construct the phylogeny. Based on multilocus sequence typing (MLST), both MSHR282 and CAM41 isolates are ST105 (red), and isolates MSHR4004 and SHCH2430 are ST849 (blue). However, whole-genome phylogenetic analysis clearly groups these strains based upon geographic origin, i.e., MSHR282 and MSHR4004 group with other Australian isolates, and CAM41 and SHCH2430 group with other Asian strains. Thus, MLST of B. pseudomallei can, in rare cases, be confounded by ST homoplasy. The scale bar represents the average number of nucleotide substitutions per site.

To gain further insight into the genetic relatedness and geographic origin of these four isolates, we performed whole-genome sequencing (WGS) using the Illumina HiSeq2000 platform (Illumina Inc., San Diego, CA). WGS data have been deposited into the NCBI SRA database with the following accession numbers: CAM41 (ERR539773, ERR539807, and ERR539841), MSHR282 (ERR298339), SHCH240 (ERR298360), and MSHR4004 (ERR298343). Single-nucleotide polymorphisms (SNPs) in the core genome were identified with SPANDx v2.3 (14) using B. pseudomallei K96243 as a reference sequence (15). Additional reference B. pseudomallei genomes were incorporated into the analysis by inclusion of simulated Illumina data using ART v2.1.8 and a quality shift of 10 (16). Using the default settings of SPANDx, 84,839 core genome SNPs were identified. Maximum likelihood phylogenetic analysis of these SNPs using RAxML (17) grouped the Australian isolates (MSHR282 and MSHR4004) with other Australian isolates; likewise, the Cambodian isolates (CAM41 and SHCH2430) grouped most closely with isolates of Asian origin (Fig. 2). To assess the effect of recombination on phylogenetic inference, recombinogenic regions were removed using altered SNP-filtering parameters with GATK (18) based on a SNP density of more than two SNPs within 300 bp or with gubbins based on default parameters (19). GATK filtering or gubbins analysis removed 37,213 (44%) or 24,216 (13.5%) SNPs, respectively, used in the phylogenetic reconstruction but did not alter geographic attribution of strains or tree topology (see Fig. S1 and S2 in the supplemental material). Overall, these findings suggest that both ST105 and ST849 convergence was a consequence of both mutation and multiple recombination events over considerable evolutionary time rather than from recent recombination involving the MLST loci (Fig. S1).

To complement the whole-genome SNP phylogeny findings, several variable genetic markers with known geographic associations were interrogated *in silico*; specifically, the virulence factors encoded by Burkholderia pseudomallei or Burkholderia mallei *bimA* (*bimA*_Bp/Bm_) (20) and *fhaB3* (21), and the BTFC (Burkholderia thailandensis-like fimbrial cluster) and YLF (Yersinia-like fimbrial cluster) loci, the latter two of which are mutually exclusive. All four isolates possessed *fhaB3* and the *bimA*_Bp_ subtype. Only the MSHR4004 isolate was positive for BTFC; all other isolates possessed the YLF allele. The *fhaB3*, *bimA*_Bp_, and YLF markers are more common in B. pseudomallei isolates of Asian origin, and *bimA*_Bm_ has yet to be observed in any Southeast Asian isolate but is present in 12% of Australian isolates (22, 23). These genotypes correlate with their expected prevalence in Australian and Southeast Asian B. pseudomallei. The *bimA*_Bm_ and BTFC loci were not observed in the two Cambodian isolates. The discordant YLF/BTFC profiles in the two ST849 isolates is highly unusual. To the best of our knowledge, this has not been reported previously in isolates with identical STs from the same geographic region. Taken together, these results support the expected geographic origin of these isolates and further consolidate the convergent nature of these STs.

We report, for the first time, two instances of B. pseudomallei isolates with identical STs from two continents. Isolate origins were resolved using whole-genome phylogenetic analysis. Although our study was limited by the availability of Cambodian B. pseudomallei isolates for comparative whole-genome phylogenetic analysis, we showed that, in both cases, shared STs between geographic regions were due to ST homoplasy. Our findings rule out recent B. pseudomallei transmission between these regions and demonstrate some limitations of MLST for source attribution of highly recombinogenic species.

## Supplementary Material

Supplemental material
