# Supplementary material for: Whole-Genome Sequencing Confirms that Burkholderia pseudomallei Multilocus Sequence Types Common to Both Cambodia and Australia Are Due to Homoplasy
Source: J Clin Microbiol. 2014 Dec 18;53(1):323–6. doi: 10.1128/JCM.02574-14 (PMC4290968; doi:10.1128/JCM.02574-14)
Supplement: Supplemental material [file supp_53_1_323__index.html]

Whole-Genome Sequencing Confirms that Burkholderia pseudomallei Multilocus Sequence Types Common to Both Cambodia and Australia Are Due to Homoplasy — Supplemental material 

# Whole-Genome Sequencing Confirms that Burkholderia pseudomallei Multilocus Sequence Types Common to Both Cambodia and Australia Are Due to Homoplasy

## Supplemental material

**Files in this Data Supplement:**

- Supplemental file 1 -

  Fig. S1 (Maximum likelihood phylogenetic tree inferred by RAxML after removal of recombinogenic regions by use of GATK SNP density filtering) and S2 (Maximum likelihood phylogenetic tree inferred by RAxML after removal of recombinogenic regions by use of gubbins)

  PDF, 343K
