# Supplementary material for: Whole-Genome Sequencing Confirms that Burkholderia pseudomallei Multilocus Sequence Types Common to Both Cambodia and Australia Are Due to Homoplasy
Source: J Clin Microbiol. 2014 Dec 18;53(1):323–6. doi: 10.1128/JCM.02574-14 (PMC4290968; doi:10.1128/JCM.02574-14)
Supplement: Supplemental material [file JCM.02574-14_zjm999093972so1.pdf]

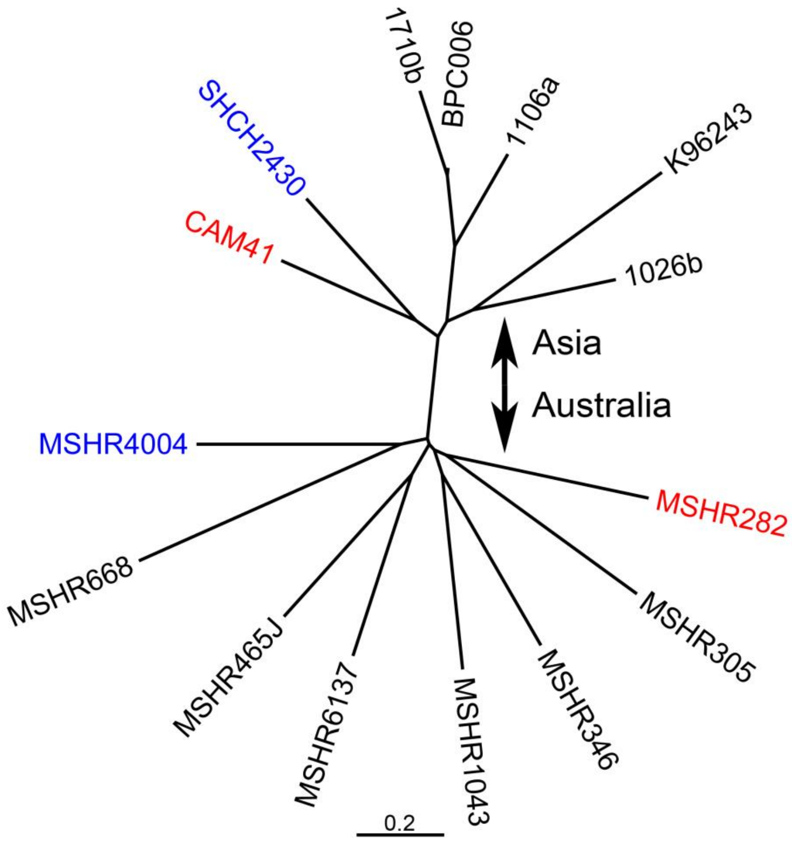

Figure S1: Maximum-likelihood phylogenetic tree inferred by RAxML (1) after removal of recombinogenic regions using GATK SNP density filtering. SNP clusters were removed by altering the GATK (2) SNP cluster filter parameter to remove clusters containing >2 SNPs per 300bp. Tree topology remains similar to the initial maximum likelihood analysis containing all SNPs. Importantly, geographical attribution of the geographical disparate isolates with the same sequence type remains identical regardless of inclusion or exclusion of recombinogenic regions. In all SPANDx (3) analyses K96243 was used as the reference genome.

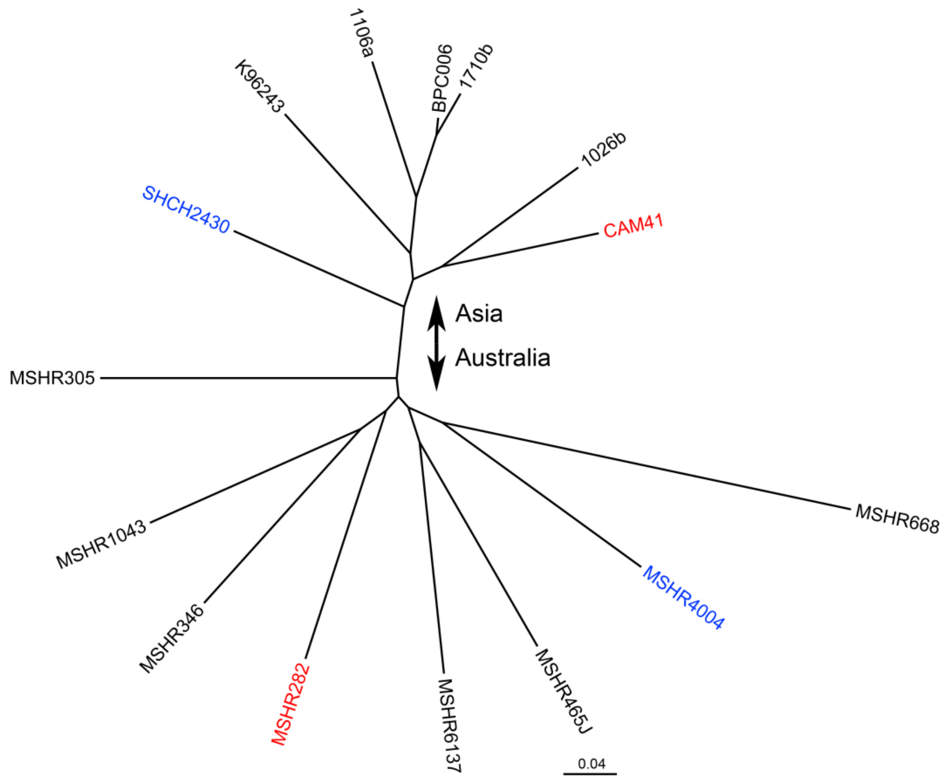

Figure S2: **Maximum-likelihood phylogenetic tree inferred by RAxML (1) after removal of recombinogenic regions using gubbins (4).** Whole genome sequence data were assembled using Velvet (5) with parameters optimized using VelvetOptimiser. Draft assemblies were improved and error-corrected with ABACAS, IMAGE and ICORN2 (6). Core-genome single-nucleotide polymorphisms (SNPs) were identified from aligned assemblies using progressiveMauve (7) before recombinogenic region identification using gubbins and subsequent inference of phylogenetic relatedness with RAxML.

1. **Stamatakis A.** 2014. RAxML version 8: a tool for phylogenetic analysis and post-analysis of large phylogenies. Bioinformatics **30:**1312-1313.
2. **McKenna A, Hanna M, Banks E, Sivachenko A, Cibulskis K, Kernytsky A, Garimella K, Altshuler D, Gabriel S, Daly M, DePristo MA.** 2010. The Genome Analysis Toolkit: a MapReduce framework for analyzing next-generation DNA sequencing data. Genome Res **20:**1297-1303.
3. **Sarovich D, Price E.** 2014. SPANDx: a genomics pipeline for comparative analysis of large haploid whole genome re-sequencing datasets. BMC Res. Notes **Accepted for publication**.
4. **Croucher NJ, Harris SR, Fraser C, Quail MA, Burton J, van der Linden M, McGee L, von Gottberg A, Song JH, Ko KS, Pichon B, Baker S, Parry CM, Lambertsen LM, Shahinas D, Pillai DR, Mitchell TJ, Dougan G, Tomasz A, Klugman KP, Parkhill J, Hanage WP, Bentley SD.** 2011. Rapid pneumococcal evolution in response to clinical interventions. Science **331:**430-434.
5. **Zerbino DR, Birney E.** 2008. Velvet: algorithms for de novo short read assembly using de Bruijn graphs. Genome Res. **18:**821-829.

6. **Swain MT, Tsai IJ, Assefa SA, Newbold C, Berriman M, Otto TD.** 2012. A post-assembly genome-improvement toolkit (PAGIT) to obtain annotated genomes from contigs. Nat. Protoc. **7:**1260-1284.
7. **Darling AE, Mau B, Perna NT.** 2010. progressiveMauve: multiple genome alignment with gene gain, loss and rearrangement. PLoS One **5:**e11147.
